# Establishing a nomogram to predict refracture after percutaneous kyphoplasty by logistic regression

**DOI:** 10.3389/fninf.2023.1304248

**Published:** 2023-12-21

**Authors:** Aiqi Zhang, Hongye Fu, Junjie Wang, Zhe Chen, Jiajun Fan

**Affiliations:** ^1^The Second Clinical Medical College of Zhejiang Chinese Medical University, Hangzhou, Zhejiang, China; ^2^The Fourth Clinical Medical College of Zhejiang Chinese Medical University, Hangzhou, Zhejiang, China; ^3^Department of Orthopedics, The Second Affiliated Hospital of Zhejiang Chinese Medical University, Hangzhou, Zhejiang, China

**Keywords:** vertebral compression fractures, percutaneous kyphoplasty, logistic regression, random forest model, anterior vertebral height

## Abstract

**Introduction:**

Several studies have examined the risk factors for post-percutaneous kyphoplasty (PKP) refractures and developed many clinical prognostic models. However, no prior research exists using the Random Forest (RF) model, a favored tool for model development, to predict the occurrence of new vertebral compression fractures (NVCFs). Therefore, this study aimed to investigate the risk factors for the occurrence of post-PKP fractures, compare the predictive performance of logistic regression and RF models in forecasting post-PKP fractures, and visualize the logistic regression model.

**Methods:**

We collected clinical data from 349 patients who underwent PKP treatment at our institution from January 2018 to December 2021. Lasso regression was employed to select risk factors associated with the occurrence of NVCFs. Subsequently, logistic regression and RF models were established, and their predictive capabilities were compared. Finally, a nomogram was created.

**Results:**

The variables selected using Lasso regression, including bone density, cement distribution, vertebral fracture location, preoperative vertebral height, and vertebral height restoration rate, were included in both the logistic regression and RF models. The area under the curves of the logistic regression and RF models were 0.868 and 0.786, respectively, in the training set and 0.786 and 0.599, respectively, in the validation set. Furthermore, the calibration curve of the logistic regression model also outperformed that of the RF model.

**Conclusion:**

The logistic regression model provided better predictive capabilities for identifying patients at risk for post-PKP vertebral fractures than the RF model.

## 1 Introduction

Osteoporotic vertebral compression fractures are one of the most prominent etiological factors that contribute to back pain, reduced daily activity levels, and increased bed rest durations in patients. This condition diminishes the quality of life and is associated with elevated mortality rates in more severe cases (Lyritis et al., 1989; Goldstein et al., 2015). Therapeutic interventions for osteoporotic vertebral compression fractures involve conservative and surgical approaches. Although conservative management provides symptom alleviation, it is burdened by the risk of malunion and the progression of kyphotic deformities, culminating in symptom recurrence (Soultanis et al., 2021). Percutaneous kyphoplasty (PKP) has garnered widespread recognition for its efficacy in addressing osteoporotic vertebral compression fractures. It has also demonstrated analgesic effects exceeding 90%, facilitating the early mobilization of patients and mitigating the incidence of complications (Li et al., 2015). Scholarly attention has been drawn to post-PKP cases with the evolution of PKP techniques, presenting with new vertebral compression fractures (NVCFs). Reports have cited NVCF occurrence rates ranging from 2 to 23% (Chen et al., 2021). Furthermore, several variables have been implicated in NVCF etiology, including age, bone mineral density (BMD), body mass index, the volume of administered bone cement, and instances of cement leakage (Bian et al., 2022; Zhang et al., 2023).

Numerous investigations have scrutinized the risk factors for post-PKP refractures and developed several clinical prognostic models (Li et al., 2021; Bian et al., 2022; Gao et al., 2023). However, most of these studies predominantly employed univariate or multivariate analyses, and the importance of the variables related to risk factors was usually not elucidated. Therefore, this ambiguity may impede clinical decision-making regarding the focal points of preventive measures post-PKP surgery. The Random Forest (RF) algorithm is a favored tool for model development and is distinguished as one of the most proficient techniques available (Iverson et al., 2008). It is also invaluable for ranking the relative importance of risk factors beyond model development, thereby assisting clinicians in pinpointing pivotal determinants for NVCF prevention. However, no prior research utilizing the RF model to predict NVCF occurrence exists. Therefore, this study aimed to compare the predictive performance of logistic regression and RF prediction models for NVCF. Specifically, this study’s objective was to select the most suitable model for predicting NVCF, providing a reference for tailoring individualized treatment plans for the early prevention of post-PKP fractures.

## 2 Materials and methods

### 2.1 Study population

We conducted a comprehensive retrospective analysis of clinical data obtained from patients who underwent PKP for single-level osteoporotic vertebral compression fractures in our healthcare institution between January 2018 and December 2021. Our inclusion criteria comprised the following: (1) the occurrence of a single-level vertebral body fracture resulting from low-energy trauma, such as a fall, which subsequently led to lower back pain or restricted mobility and subsequent PKP intervention; (2) radiographic evaluations, including X-rays and computed tomography scans, indicating the presence of vertebral compression fractures, corroborated by magnetic resonance imaging confirming recent vertebral fractures; and (3) the availability of complete clinical data. The exclusion criteria were as follows: (1) vertebral fractures caused by tumors, infections, or tuberculosis; (2) vertebral fractures resulting from high-energy trauma, such as a fall from a height or a motor vehicle accident; (3) spinal cord compression with neurological symptoms; (4) poor cardiopulmonary function rendering the patient unfit for surgery; and (5) secondary osteoporosis.

A total of 349 patients who met the inclusion criteria were included in the study. The fractured segments were distributed as follows: T5–T9, T10–L2, and L3–L5 in 31, 248, and 70 cases, respectively. The enrolled patients were randomly categorized into the training and validation groups in a 7:3 ratio. The training and validation groups were used for variable selection and model development and to assess the model’s performance, respectively.

This study was reviewed and approved by the Ethics Committee of Zhejiang Provincial People’s Hospital (QT2023051). The need for obtaining informed consent was exempted by the ethics committee because of the retrospective nature of this study.

### 2.2 Observation indicators

**Figure 1:**
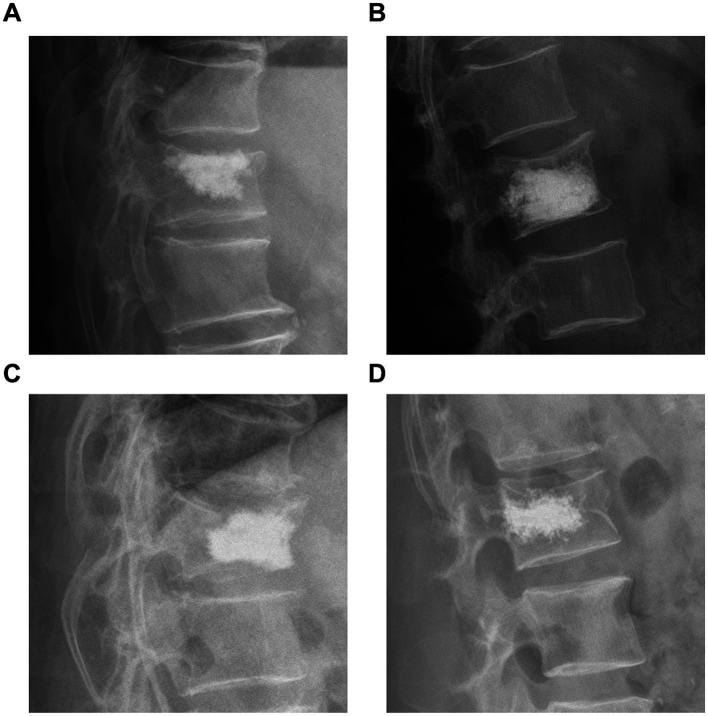
**(A)** Bone cement in contact with the upper edge of the vertebral endplate. **(B)** Bone cement in contact with the lower edge of the vertebral endplate. **(C)** Bone cement in contact with both the upper and lower edges of the vertebral endplate. **(D)** Bone cement does not contact either the upper or lower edges of the vertebral endplate.

In a retrospective analysis, we collected data on age, sex, diabetes, hypertension history, body mass index, BMD, the vertebral segment of the fracture, the average volume of bone cement used, bone cement leakage, bone cement dispersion, bone cement distribution (Figure 1), contact of bone cement with the endplate, osteoporosis treatment, spinal curvature, preoperative and postoperative Cobb angle, and preoperative and postoperative anterior vertebral height (AVH) (Table 1). Adequate dispersion of bone cement on spinal X-rays was defined as the cement crossing the midline of the vertebral body; otherwise, it was considered poor dispersion. The AVH recovery ratio was defined as the difference between postoperative and preoperative AVH divided by the average of the adjacent vertebrae to the injured vertebra. Furthermore, the Cobb angle was defined as the angle formed by the upper and lower endplates of the fractured vertebra, while the Cobb angle recovery rate was the preoperative Cobb angle divided by the postoperative Cobb angle in percentiles.

**Table 1:** Basic characteristics of clinical data.

Variables	Total (*n* = 349)	Control (*n* = 301)	NVCF (*n* = 48)	*p*
Sex, *n* (%)				0.501
Male	63 (18.1%)	56 (18.6%)	7 (14.6%)	
Female	286 (81.9%)	245 (81.4%)	41 (85.4%)	
Age (years)	74.42 ± 9.70	73.85 ± 9.68	78.04 ± 9.11	0.005
BMI (kg/m^2^)	22.54 ± 3.7	22.75 ± 3.69	21.22 ± 3.83	0.012
BMD	−2.56 ± 1.30	−2.46 ± 1.27	−3.23 ± 1.31	<0.001
History of hypertension, *n* (%)				0.177
Yes	177 (50.7%)	157 (52.2%)	20 (41.7%)	
No	172 (49.3%)	144 (47.8%)	28 (58.3%)	
History of diabetes, *n* (%)				0.830
Yes	62 (17.8%)	54 (17.9%)	8 (16.7%)	
No	287 (82.2%)	247 (82.1%)	40(83.3%)	
Augmentation segment, *n* (%)				<0.001
T5–9	31 (8.9%)	19 (6.3%)	12 (25.0%)	
T10–L2	248 (71.1%)	217 (72.1%)	31 (64.6%)	
L3–5	70 (20.0%)	65 (21.6%)	5 (10.4%)	
Bone cement dosage (mL)	4.89 ± 1.24	4.90 ± 1.25	4.82 ± 1.16	0.656
Bone cement leakage, *n* (%)				0.006
Yes	89 (25.5%)	69 (22.9%)	20 (41.7%)	
No	260 (74.5%)	232 (77.1%)	28(58.3%)	
Bone cement dispersion, *n* (%)				0.358
Yes	340 (97.4%)	294 (97.7%)	46 (95.8%)	
No	9 (2.6%)	7(2.3%)	2 (4.2%)	
Bone cement distribution, *n* (%)				0.002
Shape A	26 (7.4%)	23 (7.6%)	3 (6.3%)	
Shape B	70 (20.1%)	64 (21.3%)	6 (12.5%)	
Shape C	183 (52.4%)	164 (54.5%)	19 (39.6%)	
Shape D	70 (20.1%)	50 (16.6%)	20 (41.7%)	
Contact with the endplates, *n* (%)				0.009
Yes	15 (4.3%)	9 (3.0%)	6 (12.5%)	
No	334 (95.7%)	292 (97.0%)	42 (87.5%)	
Anti-osteoporotic treatment, *n* (%)				0.004
Yes	155 (44.4%)	143 (47.5%)	12 (25.0%)	
No	194 (55.6%)	158 (52.5%)	36 (75.0%)	
Scoliosis, *n* (%)				0.141
Yes	120 (34.4%)	99 (32.9%)	21 (43.8%)	
No	229 (65.6%)	202 (67.1%)	27 (56.2%)	
Preoperative AVH (mm)	18.80 ± 6.11	19.60 ± 5.86	13.79 ± 5.29	<0.001
Postoperative AVH (mm)	22.30 ± 5.35	22.85 ± 5.21	18.86 ± 5.02	<0.001
AVHRR, *n* (%)	14.35 ± 13.22	12.98 ± 13.13	22.95 ± 10.28	<0.001
Preoperative Cobb angle (°)	7.34 ± 6.33	7.11 ± 6.28	8.81 ± 6.58	0.117
Postoperative Cobb angle (°)	6.78 ± 6.09	6.54 ± 6.06	8.30 ± 6.08	0.083
Cobb angle restoration (%)	0.34 ± 0.27	0.32 ± 0.28	0.42 ± 0.23	0.007

AVH, anterior vertebral height; AVHRR, anterior vertebral height recovery ratio; BMD, bone mineral density; BMI, body mass index; NVCF, new vertebral compression fracture.

### 2.3 Surgical methods

Patients were placed in the prone position, and a C-arm X-ray machine facilitated anteroposterior and lateral fluoroscopic localization of the bilateral pedicles of the affected vertebrae. Following standard disinfection and draping procedures, local infiltration anesthesia was administered at the puncture sites of the bilateral pedicles. The procedure was initiated on the left side with an approximately 1 cm longitudinal incision at the left pedicle’s projection. Next, a puncture was carefully performed at the 10 o’clock position after dissecting the skin and subcutaneous tissues, with attention to sagittal and angulation angles. Under fluoroscopic guidance using the C-arm X-ray machine in the lateral position, confirmation of the puncture needle’s central alignment within the pedicle was ensured. Subsequently, the puncture needle was withdrawn, and a guiding needle was introduced. A working cannula was placed approximately 0.5 cm anterior to the vertebral body’s posterior margin, replacing the guiding needle. A tamp was inserted, followed by an inflated balloon with a contrast medium to the appropriate pressure. Next, the balloon was extracted after the contrast medium was removed. The procedure was repeated on the right side, with a puncture made at the 2 o’clock position. Slow injection of bone cement was conducted under fluoroscopic guidance, establishing an effective mechanical column. Finally, the cannula was removed following cement solidification, and cement injection was promptly halted in cases of cement leakage during the procedure.

### 2.4 Postoperative management and refracture assessment

All patients received calcium supplements and active vitamin D combined with zoledronic acid or denosumab as standard treatment postoperatively. Thoracolumbar braces were worn for 3 months, and postoperative spinal X-rays, including anteroposterior and lateral views, were obtained. Spinal magnetic resonance imaging was employed for patients suspected of having vertebral refractures. The diagnostic criteria for post-PKP refractures encompassed the following: (1) reappearance of lower-back pain or restricted lumbar mobility post-PKP; (2) spinal magnetic resonance imaging indicating a new fracture characterized by low and high signals on T1- and T2-weighted imaging, respectively; and (3) the distinct identification of the fractured vertebra from the preoperative one.

### 2.5 Statistical analysis

Data analysis and graphical representation utilized IBM SPSS Statistics for Windows, version 26 (IBM Corp., Armonk, N.Y., United States) and R 4.22 software. Continuous variables were expressed as means ± standard deviations and compared using independent samples t-tests. Categorical variables were presented as percentages and compared using chi-square tests. Lasso regression aided in the selection of risk factors. The parameters selected by Lasso regression informed the construction of logistic regression and RF predictive models. Lasso regression analysis was conducted using the “glmnet” package. Given its non-intuitive output, variable importance was employed to elucidate the significance of each predictive factor in the RF model. The “randomForest” package was employed for the RF model and variable importance analysis. Additionally, the receiver operating characteristic curve was constructed, and the area under the curve value was calculated to assess sensitivity and specificity. The receiver operating characteristic curve was generated using the “pROC” package. A calibration plot was generated to examine the performance characteristics of the predictive model. Furthermore, the calibration curve was plotted using the “ggplot2” package, and statistical significance was considered at *p* < 0.05.

## 3 Results

A cohort of 349 patients was examined, with 48 (13.7%) experiencing refractures. The sex distribution in the control group was 56 (18.4%) males and 245 (81.6%) females, with a mean age and BMD of 73.85 ± 9.68 years and − 2.46 ± 1.27, respectively. In contrast, the refracture group included 7 (14.6%) males and 41 (85.4%) females, with a mean age and BMD of 78.04 ± 9.11 years and − 3.23 ± 1.31, respectively. Moreover, osteoporosis treatment was more prevalent in the control group than in the refracture group. The vertebral height recovery rate was significantly lower in the control group than in the refracture group. Table 1 summarizes the additional baseline characteristics of the enrolled patients.

### 3.1 Key variables

**Figure 2:**
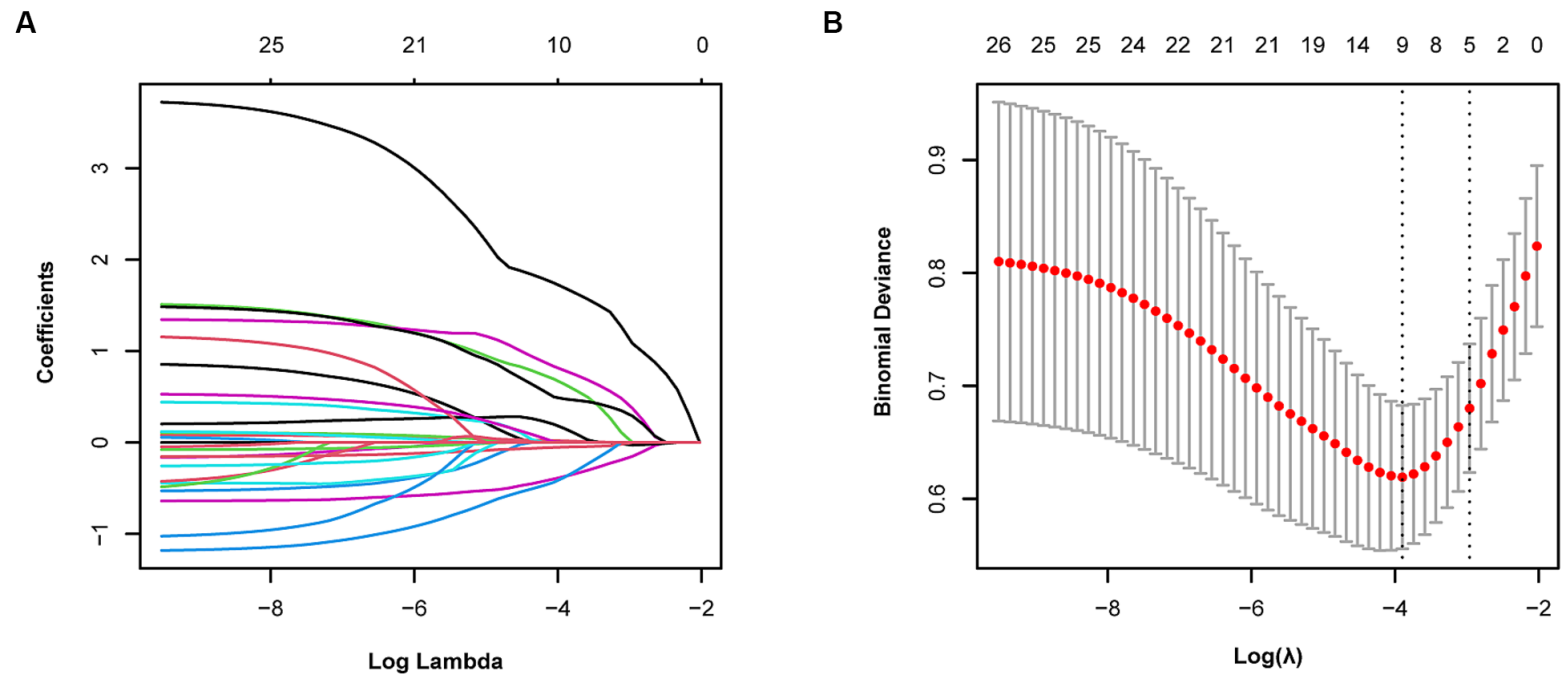
Variable selection using Lasso regression. **(A)** Coefficient fluctuations for variables. **(B)** Lasso regression λ determined through tenfold cross-validation.

We observed coefficient variations for each variable using Lasso regression for variable selection (Figure 2A). Additionally, we identified an optimal model with superior performance and minimal variables at λ = 0.051 (ln λ = −2.98) employing tenfold cross-validation (Figure 2B). The selected variables comprised BMD, fracture location, bone cement shape, preoperative vertebral height, and vertebral height recovery rate.

### 3.2 Establishment of logistic regression and RF models

We further established a logistic regression model based on the parameters identified via Lasso regression (Table 2), and this model demonstrated training set metrics of accuracy, specificity, sensitivity, and F1 score at 0.780, 0.800, 0.777, and 0.859, respectively. The training set area under curve, accuracy, specificity, sensitivity, and F1 score were 0.825, 0.846, 0.822, and 0.892, respectively (Table 3).

**Table 2:** Parameters for the logistic regression model.

Variables	B	Z	OR (95% CI)	*p*
BMD	−0.549	−1.579	0.578 (0.292–1.142)	0.114
Shape D	1.364	2.791	3.912 (1.500–10.201)	0.005
Augmentation segment (T5–T9)	0.853	1.384	2.347 (0.700–7.865)	0.167
Preoperative AVH	−0.071	−1.551	0.932 (0.851–1.019)	0.121
AVHRR	2.217	3.344	8.391 (2.412–29.186)	0.001

AVH, anterior vertebral height; AVHRR, anterior vertebral height recovery ratio; BMD, bone mineral density; CI, confidence interval; OR, odds ratio.

**Table 3:** Metrics for the training and validation sets.

	Logistic Regression		Random Forest	
	Training dataset	Validation dataset	Training dataset	Validation dataset
Accuracy	0.780	0.825	0.943	0.757
Specificity	0.800	0.846	0.971	0.615
Sensitivity	0.777	0.822	0.938	0.778
F1 Score	0.859	0.892	0.954	0.687

**Figure 3:**
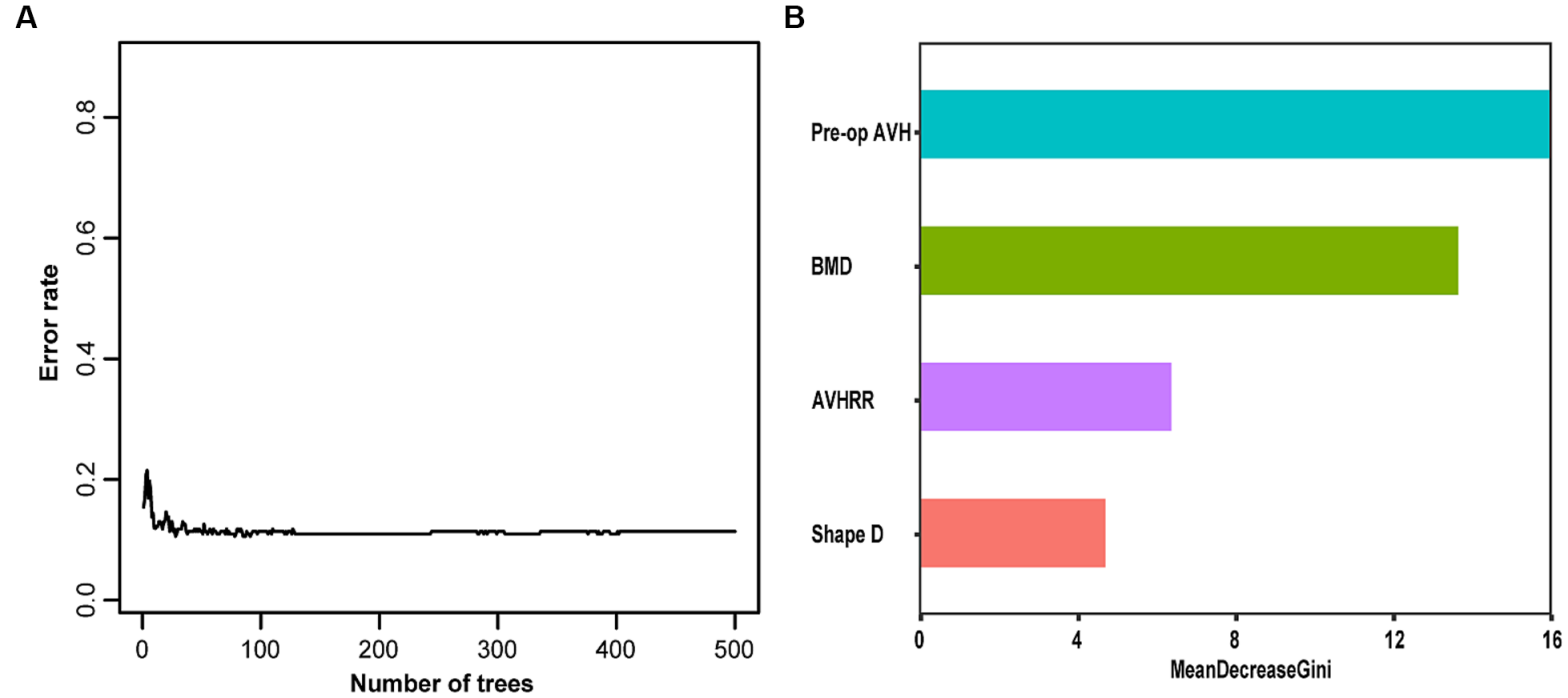
**(A)** Random Forest error rate. **(B)** Out-of-bag variable importance ranking. AVH, anterior vertebral height; AVHRR, anterior vertebral height recovery ratio; BMD, bone mineral density.

We constructed an RF model in the training set using the same set of variables. With an ntree value of 28, the out-of-bag error rate was low at 11.38% (Figure 3A). The training set exhibited accuracy, specificity, sensitivity, and F1 score metrics of 0.943, 0.971, 0.938, and 0.954, respectively, whereas the validation set metrics showed accuracy, specificity, sensitivity, and F1 score of 0.757, 0.615, 0.778, and 0.687, respectively (Table 3) (Figure 3B).

### 3.3 Performance of logistic regression and RF models

**Figure 4:**
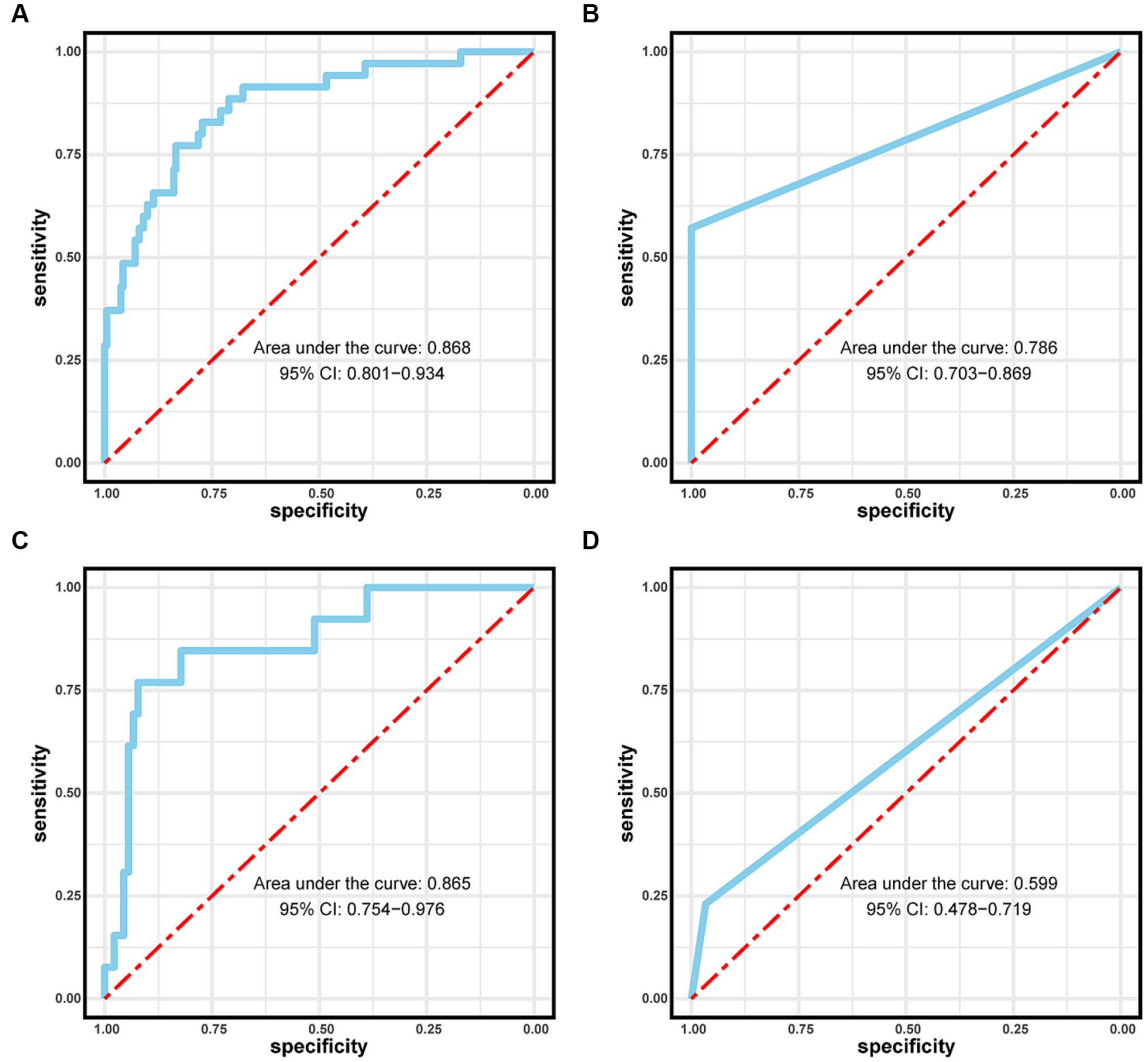
Area under the curves (AUCs) for the logistic regression model in the **(A)** training and **(C)** validation sets. AUCs for the Random Forest model in the **(B)** training and **(D)** validation sets.

**Figure 5:**
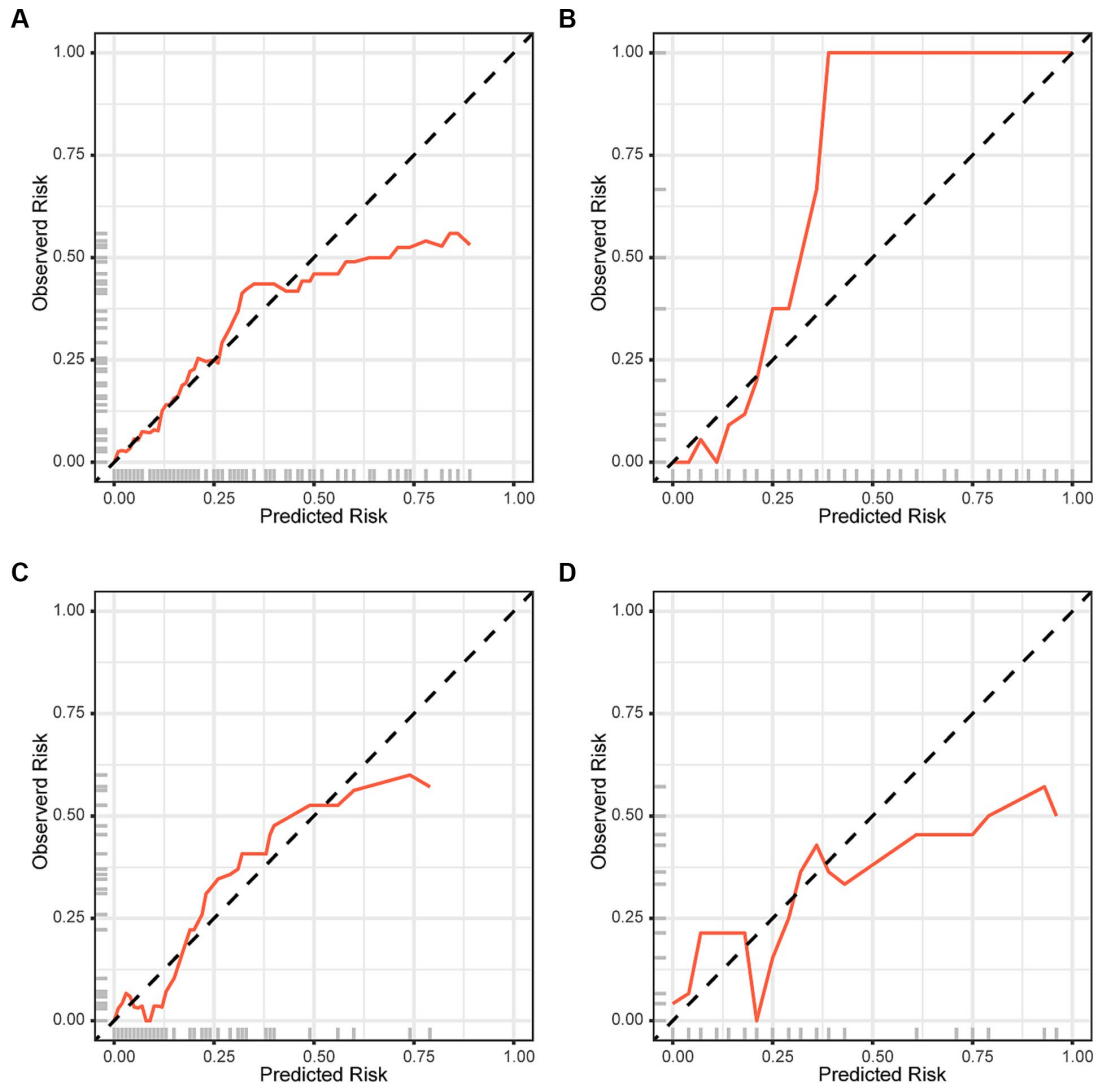
Calibration curves for the Logistic regression model in the **(A)** training and **(C)** validation sets. Calibration curves for the Random Forest model in the **(B)** training and **(D)** validation sets.

The performance of the logistic regression and RF models was evaluated using receiver operating characteristic and calibration curves. The area under the curve for the logistic regression and RF models within the training set were 0.868 and 0.786, respectively, but were 0.786 and 0.599 in the validation set, respectively (Figure 4). The logistic regression model consistently outperformed the RF model in the training and validation sets. Calibration curves were subsequently constructed, and the logistic regression model outperformed the RF model in both the training and validation sets (Figure 5).

### 3.4 Nomogram development

**Figure 6:**
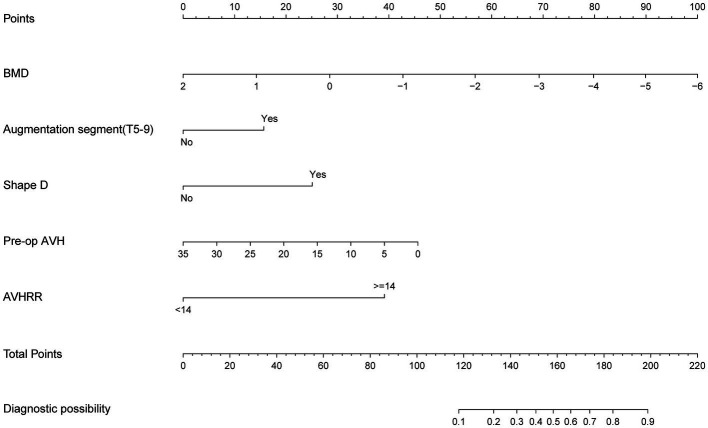
Nomogram for predicting post-PKP refractures. AVH, anterior vertebral height; AVHRR, anterior vertebral height recovery ratio; BMD, bone mineral density; PKP, percutaneous kyphoplasty.

We selected the logistic regression model to predict the recurrence risk of NCVF post-PKP. Next, we transformed the complex mathematical model into a graphical format for improved clinical applicability. For instance, if a patient is male, has a T9 vertebral fracture, a BMD of −3.0, vertebral height recovery >14%, a bone cement distribution shape of D, and preoperative vertebral height of 20 mm, their total score would be approximately 169, indicating a risk of approximately 64% for developing NCVF post-PKP (Figure 6).

## 4 Discussion

PKP provides rapid relief from severe pain that is difficult to manage conservatively and effectively reduces cement leakage due to the presence of an expandable balloon (Chen et al., 2018; Hu et al., 2018). It allows for the rapid restoration of spinal lordosis, thereby improving biomechanical stability (Lee et al., 2014; Cao et al., 2020). Furthermore, PKP enables early mobilization, reducing bed rest time and the risk of complications. Common post-PKP complications include NCVF, pulmonary embolism, superior vena cava injury, and postoperative back pain, with NCVF being particularly common and severely affecting patients’ quality of life (Li et al., 2021; Yu et al., 2021; Zou et al., 2021). However, some patients experienced multiple NVCFs, which significantly undermined the confidence in these patients’ recovery and increased the economic and time costs associated with their treatment. Therefore, analyzing the risk factors for NVCF post-PKP and establishing a predictive model to assess this risk is essential. This can enhance patient satisfaction during the treatment process and contribute to the effective use of healthcare resources.

This study involving 349 patients resulted in the development of a nomogram based on several variables selected using Lasso regression. The predictive model incorporates five clinical assessment indicators as follows: BMD, bone cement distribution (shape D), T5–T9 vertebral fractures, preoperative vertebral height, and vertebral height recovery rate. Preoperative vertebral height and BMD evidently play a crucial role in the occurrence of post-PKP fractures (Figure 3B). Therefore, this may become a key focus for clinicians in the management of high-risk patients post-PKP in the future. These indicators allow for the evaluation of individual NCVF risk, assigning scores based on each variable’s contribution to the outcome and summing these scores. Drawing a vertical line at the corresponding total score position intersects with the line representing the risk of recurrence.

Lasso regression can address the issue of multicollinearity among variables, which is why this study selected it for variable selection. The logistic regression and RF models showed similar performance on the training dataset; however, the RF model exhibited poorer performance on the validation dataset. Therefore, a nomogram was developed in this study based on the logistic regression model. Previous research has also compared the predictive capabilities of both models for binary classification issues, and the results consistently indicate that the RF model performs poorly (Wang et al., 2022). This aligns with our study findings. Therefore, this study does not recommend using the RF model to predict the risk of post-PKP refracture.

**Figure 7:**
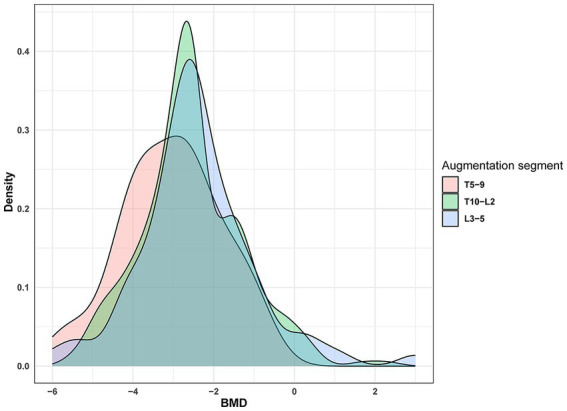
Distribution of vertebral fracture sites. BMD, bone mineral density.

Osteoporosis is a skeletal disorder characterized by reduced bone mass and microstructural deterioration, increasing bone fragility and fracture susceptibility (Sambrook and Cooper, 2006). BMD reflects the severity of osteoporosis, exhibiting a strong correlation with fracture occurrence (Black et al., 2020). Reduced BMD is typically associated with decreased bone formation capacity, vertebral height loss, and necrotic bone formation (Qi et al., 2021). Furthermore, changes in BMD effectively predict vertebral fractures, with increased BMD almost certainly lowering the risk of vertebral fractures (Bouxsein et al., 2019). Bisphosphonates and denosumab are first-line treatments for osteoporosis, effectively increasing BMD (Reid and Billington, 2022). Additionally, anti-osteoporosis treatment significantly reduces fracture risk, and the distribution of bone cement within the vertebra can impact NCVF occurrence. However, poor cement distribution may provide inadequate support, leading to NCVF. Ideally, bone cement should be in contact with the upper and lower vertebral endplates, which better restores vertebral strength and reduces the risk of NCVF (Tan et al., 2020). Our study found that patients with T5–T9 vertebral fractures were more prone to NCVF than those with T10–L2 fractures. T10–L2 is susceptible to compression fractures because it represents the junction between thoracic kyphosis and lumbar lordosis, concentrating trunk mobility stress. Clinical data review revealed that patients with T5–T9 vertebral fractures had lower BMD levels (−3.04 ± 1.24) than those with T10–L2 and L3–L5 fractures (−2.54 ± 1.26 and − 2.42 ± 1.45, respectively) (Figure 7). Furthermore, patients with T5–T9 vertebral fractures had lower BMD, suggesting a potential association between lower BMD and NCVF occurrence.

Previous studies have emphasized the significance of the AVH recovery ratio in post-PKP refracture (Niu et al., 2015; Li et al., 2018). Specifically, Lin et al. (2008) reported a significant association between AVH recovery ratio and post-PKP refracture. Excessive restoration of vertebral height may lead to increased tension in the adjacent soft tissues, resulting in elevated mechanical loads on the vertebrae or increased instability in the fractured segment (Kim et al., 2004, 2010; Niu et al., 2015) and can elevate intra-vertebral pressure, promoting bone necrosis (Heo et al., 2009). Furthermore, our model incorporated the preoperative AVH as a risk factor. The risk of post-PKP refracture was found to increase with decreasing AVH. Low AVH may also indicate significant trabecular compression within the fractured vertebra, leading to increased internal pressure, which could hinder the adequate penetration of cement (Yu et al., 2018; Zhang et al., 2023). Notably, the primary goal of PKP is pain relief in vertebral fractures; therefore, the excessive pursuit of vertebral height restoration may not always be necessary.

Previous literature has reviewed the risk factors for refracture and established predictive nomograms with excellent performance, providing valuable guidance for clinical practice (Li et al., 2021; Ju and Liu, 2023; Zhang et al., 2023). Indeed, it should be emphasized that the use of Lasso regression and multivariate logistic regression for variable selection in these studies is one of the contributing factors to the disparity in selected variables. The dataset size used to build the nomogram and the variables of interest for researchers also play a role. Consequently, it is anticipated that different datasets and risk factors may yield diverse sets of refracture risk factors in future studies. We recommend that researchers make every effort to employ consistent methods for variable selection and use the same set of variables to mitigate the impact of the factors mentioned above on study outcomes. Notably, this approach can enhance the credibility and comparability of research findings.

This study had some limitations. First, it was a retrospective study, which may have introduced selection bias and data loss. Second, the nomogram has not been externally validated in other regions or multi-center settings. Therefore, we aim to validate the nomogram’s performance with larger samples and multi-center data in future research.

In conclusion, we established a nomogram for predicting post-PKP refracture based on a logistic regression model. This nomogram can assist in screening high-risk patients for post-PKP refracture. Therefore, clinicians can use the predicted risk of recurrence to develop personalized follow-up and treatment plans.

## Data availability statement

The raw data supporting the conclusions of this article will be made available by the authors, without undue reservation.

## Ethics statement

The studies involving humans were approved by the Ethics Committee of Zhejiang Provincial People's Hospital. The studies were conducted in accordance with the local legislation and institutional requirements. Written informed consent for participation was not required from the participants or the participants' legal guardians/next of kin in accordance with the national legislation and institutional requirements.

## Author contributions

AZ: Data curation, Methodology, Writing – original draft, Writing – review & editing. HF: Data curation, Investigation, Methodology, Writing – review & editing. JW: Methodology, Software, Supervision, Writing – original draft. ZC: Writing – review & editing. JF: Funding acquisition, Investigation, Methodology, Supervision, Writing – review & editing.
